# Mathematical modelling and projecting the second wave of COVID-19 pandemic in Europe

**DOI:** 10.1136/jech-2020-215400

**Published:** 2021-02-16

**Authors:** Qinyue Zheng, Xinwei Wang, Chunbing Bao, Zhongren Ma, Qiuwei Pan

**Affiliations:** 1 School of Management, Shandong Key Laboratory of Social Supernetwork Computation and Decision Simulation, Shandong University, Jinan, Shandong, China; 2 Department of Engineering Mechanics, State Key Laboratory of Structural Analysis for Industrial Equipment, Dalian University of Technology, Dalian, Liaoning, China; 3 Biomedical Research Center, Northwest Minzu University, Lanzhou, Gansu, China; 4 Department of Gastroenterology and Hepatology, Erasmus MC-University Medical Center, Rotterdam, South Holland, The Netherlands

**Keywords:** infection, public health, social activities

A second wave of the COVID-19 pandemic has spurred in most of the European countries since the summer of 2020, and currently it remains uncertain when and how this can be fully controlled.^1^ In this study, we aim to nowcast and forecast the possible development of the second COVID-19 wave in representative European countries including Spain, France and the Netherlands by mathematical modelling.

**Figure 1:**
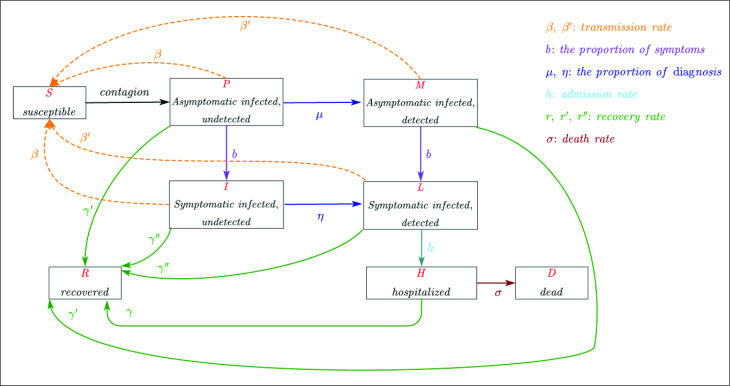
Graphical scheme representing the interactions among different compartments in the SPMILHRD model. In the mathematical model, S: susceptible, uninfected; P: infected, infectious, undetected, no symptom; M: infected, infectious, detected, no symptom; I: actively infected, infectious, undetected, with symptom; L: actively infected, infectious, detected, with symptom; H: actively infected, hospitalised, ailing, with severe symptom, life-threatening, quarantined; R: recovered or healed; D: dead; β: transmission rate from infected to susceptible individuals; b: the proportion of asymptomatic carriers developed symptoms; μ: the proportion of asymptomatic carriers who were detected; η: the proportion of symptomatic infections who were detected; h: hospital admission rate; γ: recovery rate; σ: death rate.

**Figure 2:**
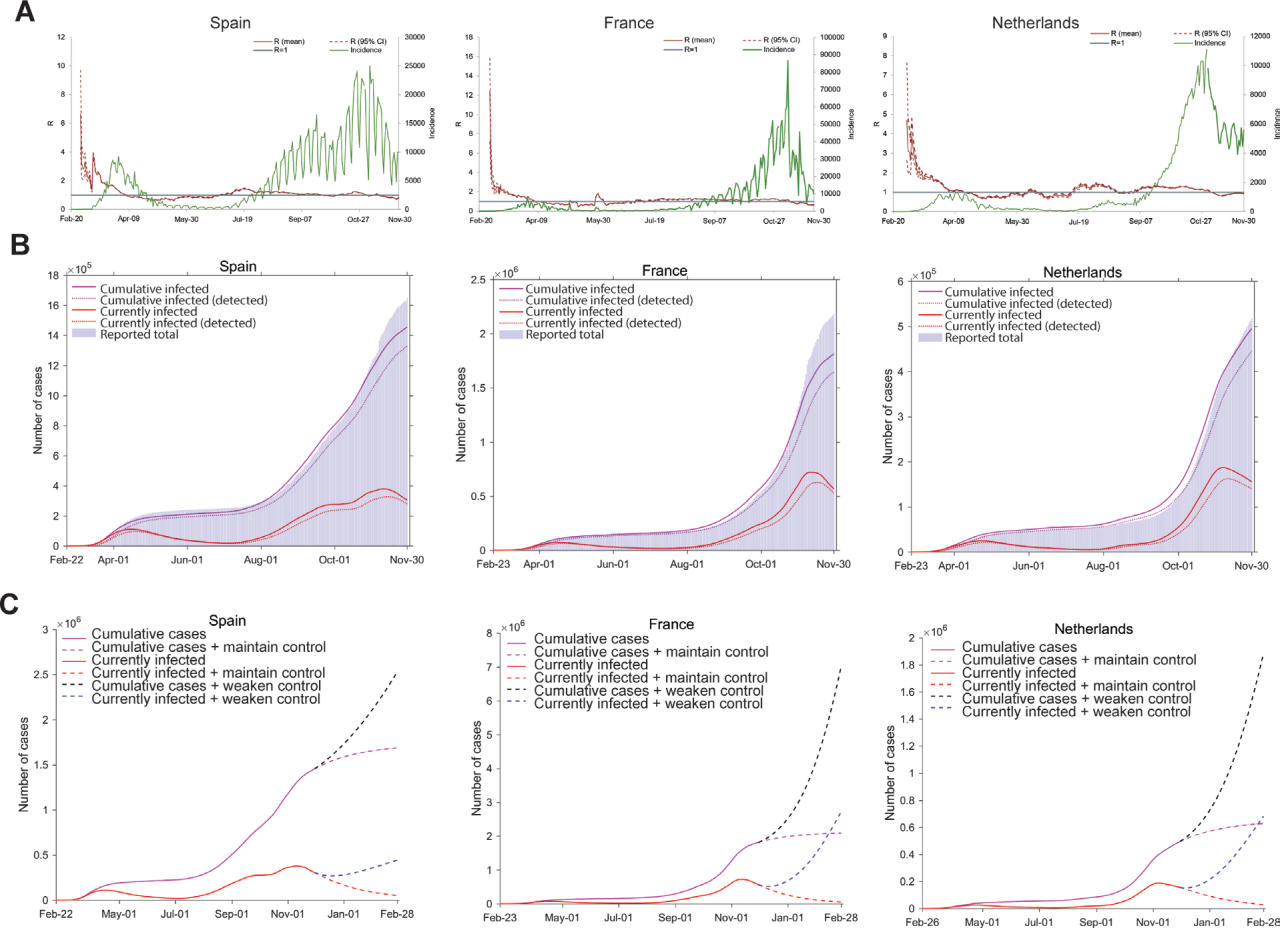
The time-varying reproduction number estimation, epidemic simulation and second wave projecting. (A) The simulation is based on incorporating *R*_*t*_ in the SPMILHRD model. Other constant parameters were defined by previous literature. Incidence curve represents the number of daily new confirmed cases reported by the WHO. The timeframe of incidence ended on 30 November 2020. The estimation of *R*_*t*_ requires the observation of incident cases over the entire window, and thus it can only be obtained at the end of that window. (B) The total or detected cumulative cases and the total or detected ongoing infections were estimated for Spain, France and the Netherlands from 20 February to 30 November 2020. The modelled results were fitted with reported real-world data. (C) Two scenarios were simulated, including maintaining the current level of control measures or weakening measures as reverting to the average level in October. The total or detected cumulative cases and total or detected ongoing infections were projected from 1 December 2020 to 28 February 2021. The shadow parts represent the 95% CI. Real-world data source: https://covid19.who.int/.

We adopted a SPMILHRD model based on the modification of the classic epidemic compartmental model SEIR (online supplemental methods; figure 1). How rapid an epidemic can spread largely depends on the reproductive number *R. R* must be below 1 to stop an outbreak. The transmission dynamics of COVID-19 can vary tremendously depending on the effectiveness of control measures. To better understand the epidemic spread, we thus estimated the time-varying reproduction number *R_t_* based on real-word reported data (figure 2A). By incorporating *R*_*t*_ in the SPMILHRD model, we recapitulated the COVID-19 epidemics in three representative European countries including Spain, France and the Netherlands (figure 2B). After the outbreaks at the end of February, control measures including lockdown and social distancing were implemented in these countries,^2^ resulting in gradual reduction of *R*_*t*_ until below 1 during April and May and the control of local epidemics. However, we observed the time-varying reproduction number has continuously exceeded 1 since 22 June in Spain, 26 June in France and 09 July in the Netherlands, with the rising of incident cases. Enhanced control measures were subsequently implemented resulting in reduction of *R*_*t*_ value until below 1 in November. The overall dynamics of epidemic spread are very similar in these three European countries.^3^ Our nowcasting on the estimated total or detected cumulative cases and the total or detected ongoing infections of the second wave by 30 November 2020 are well in line with the reported real-world cases from these three countries (figure 2B).

10.1136/jech-2020-215400.supp1 Supplementary data

We next forecasted the possible development of the second COVID-19 wave from 1 December 2020 to 28 February 2021 (figure 2C). In reality, a prolonged ‘lockdown’ is hardly possible to be implemented in Europe, in particular given that Christmas and New Year festivals are approaching. If control measures would be weakened with the transmission rate, for example, reverting to the situation as in October, the total infections would reach 1.74 times in Spain, 3.87 times in France, 3.81 times in the Netherlands after 3 months as compared with the case numbers on 30 November (figure 2C). Furthermore, the rate of detecting infected cases would be reduced, which poses more challenges for controlling the transmission.

Of note, the peak of daily new confirmed cases in the second wave is much higher than the one in the first wave in the spring of 2020 in Europe, and these differences are about 10 times in France and the Netherlands. Thus, reinforcing restriction measures is urgently required to mitigate the second wave in Europe. If the level of current measures can be maintained, the probable cumulative infections would be reduced by 33.2% (95% CI 32.9% to 33.4%) in Spain, 70.2% (95% CI 70.0% to 70.3%) in France and 66.7% (95% CI 66.3% to 67.0%) in the Netherlands in the coming 3 months since 1 December, as compared with the scenario of weakened control measures. In total, 7 million infections would be avoided in these three countries (figure 2C). Our forecasting should serve as an urgent wake-up call to the authorities and the general public to take swift actions in responding to this second wave in Europe.
