# Supplementary material for: Mathematical modelling and projecting the second wave of COVID-19 pandemic in Europe
Source: J Epidemiol Community Health. 2021 Feb 16;75(6):601–3. doi: 10.1136/jech-2020-215400 (PMC8142418; doi:10.1136/jech-2020-215400)
Supplement: Supplementary data [file jech-2020-215400supp001.pdf]

Supplementary information to

Mathematical modeling and projecting the second wave of COVID-19 pandemic in Europe

Qinyue Zheng[1], Xinwei Wang[2], Chunbing Bao[1], Zhongren Ma[3*], Qiuwei Pan[3,4*]

1. School of Management, Shandong Key Laboratory of Social Supernetwork Computation and Decision Simulation, Shandong University, Jinan 250100, China.

2. Department of Engineering Mechanics, State Key Laboratory of Structural Analysis for Industrial, Equipment, Dalian University of Technology, Dalian 116024, China.

3. Biomedical Research Center, Northwest Minzu University, Lanzhou, China.

4. Department of Gastroenterology and Hepatology, Erasmus MC-University Medical Center, Rotterdam, The Netherlands.

***Correspondence:**

Zhongren Ma (mzr@xbmu.edu.cn) or Qiuwei Pan (q.pan@erasmusmc.nl), Biomedical Research Center, Northwest Minzu University, No.1, Xibei Xincun, Lanzhou, 730030, China.

Methods

In Europe, the first confirmed cases of severe acute respiratory syndrome coronavirus 2 (SARS-COV-2) was reported in France on January 25, 2020 (1). Subsequently, outbreaks were emerged across different European countries in February and eventually became an epicenter in March (2). European countries were under lockdown one after another, with implementing strict social distance measures and extensive screening to contain the outbreak. When the case growth curve gradually flattened, the restriction measures were successively weakened in particular during the summer (3). This has triggered the concern of a possible second wave. Indeed, the incidence of new cases has suddenly risen sharply since the end of July in Spain, France, Belgium and Netherlands, and the number of new infections in Europe is escalating (4).

Epidemiological dynamic model： Predictive mathematical compartment models are widely used to understand the epidemic features and to evaluate effective strategies for control measures (5). Advanced models with more complex epidemiological compartments have been proven to accurately portray the dynamic spread of COVID-19 (6-10). High viral loads of SARS-CoV-2 were found in patients showing no/mild symptoms (6). Hence, asymptomatic carries may play a major role in transmission but underestimated (7). Importantly, most of the undetected but infected individuals are asymptomatic and are not quarantined, largely sustaining the outbreak (8).

Our model, named SPMILHRD, discriminates different severities of the disease and the status of detection. The total population is partitioned into eight epidemiological compartments (Fig. 1). The time-varying transmission rates in different countries were estimated by the time-varying reproduction number based on reported data (11), and other parameters were defined by previous literatures (8, 12). The initial values of detected cases were referred to the confirmed cases reported in World Health Organization (WHO) website. The initial values of the hospitalized, the recovered, the dead were zero.

The dynamical system describes the evolution of the subpopulations overtime:

BMJ Publishing Group Limited (BMJ) disclaims all liability and responsibility arising from any reliance placed on this supplemental material which has been supplied by the author(s)

$$
\begin{aligned}
&\dot{S} = -\beta(P+I)\frac{S}{N} - \beta' L + M)\frac{S}{N} \\
&\dot{P} = \beta(P+I)\frac{S}{N} + \beta'(L+M)\frac{S}{N} - \mu P - bP - \gamma' P \\
&\dot{M} = \mu P - bM - \gamma' M \\
&\dot{I} = bP - \eta I - \gamma'' I \\
&\dot{L} = \eta I + bM - hL - \gamma'' L \\
&\dot{H} = hL - \gamma H - \sigma H \\
&\dot{R} = \gamma H + \gamma'(P+M) + \gamma''(I+L) \\
&\dot{D} = \sigma H \\
&\dot{N} = -\sigma H
\end{aligned}
\qquad (1)
$$

Time-varying reproduction number: R_t represents the time-varying number of secondary cases generated by per infected individual. R_t infers the interventions and human-to-human transmission changes comparing with the basic reproduction number R_0 (13). The dividing line between sustaining transmission and epidemic decline is R_t=1. Here, near-real-time R_t estimation was implemented in R package EpiEstim (14) where the mean serial interval was assumed as 4.60 days and standard deviation as 5.55 (9, 13). This method has been well-suited in various COVID-19 studies (10, 15).

Scope, timeframe and data sources: We investigated the COVID-19 epidemiology of Spain, France and Netherlands using the reported data (11). The timeframe of R_t estimation and epidemic recapitulation started from February 20 and ended on November 30, 2020. The time window of COVID-19 spread prediction is three months. The key parameters, such as recovery rate, hospital admission rate, mortality rate, were assumed to be similar in the three countries including Spain, France and Netherlands.

References

1. World Health Organization. Novel Coronavirus (2019-nCoV): SITUATION REPORT - 5. 2020. https://www.who.int/docs/default-source/coronaviruse/situation-reports/20200125-sitrep-5-2019-ncov.pdf?sfvrsn=429b143d_8. Accessed January 25, 2020
2. World Health Organization. Europe is now the ‘epicenter’ of the coronavirus pandemic, WHO says. 2020. https://www.cnbc.com/2020/03/13/europe-is-now-the-epicenter-of-the-coronavirus-pandemic-who-says.html. Accessed December 01, 2020
3. World Health Organization.. Coronavirus infections havent spiked since europe loosened lockdowns there are many theories about why. 2020. https://www.washingtonpost.com/world/2020/06/05/coronavirus-infections-havent-spiked-since-europe-loosened-lockdowns-there-are-many-theories-about-why/ Accessed December 01, 2020
4. World Health Organization.. Coronavirus disease (COVID-19): Situation Report - 201. 2020. https://www.who.int/docs/default-source/coronaviruse/situation-reports/20200808-covid-19-sitrep-201.pdf?sfvrsn=121bb855_2. Accessed August 08, 2020
5. Kermack WO, McKendrick AG. A contribution to the mathematical theory of epidemics. Proceedings of the royal society of london. Series A, Containing papers of a mathematical and physical character. 1927;115(772):700-21. doi: 10.1098/rspa.1927.0118
6. Zou L, Ruan F, Huang M, et al. SARS-CoV-2 Viral Load in Upper Respiratory Specimens of Infected Patients. The New England Journal of Medicine. 2020;382(12):1177-9. doi:10.1056/NEJMC2001737
7. Gatto M, Bertuzzo E, Mari L, et al. Spread and dynamics of the COVID-19 epidemic in Italy: Effects of emergency containment measures. Proceedings of the National Academy of Sciences of the United States of America. 2020;117(19):10484-91. doi:10.1073/PNAS.2004978117
8. Giordano G, Blanchini F, Bruno R, et al. Modelling the COVID-19 epidemic and implementation of population-wide interventions in Italy. Nature Medicine. 2020;26(6):1-6. doi:10.1038/S41591-020-0883-7
9. You C, Deng Y, Hu W, et al. Estimation of the time-varying reproduction number of COVID-19 outbreak in China. International Journal of Hygiene and Environmental Health. 2020;228. doi:10.1016/J.IJHEH.2020.113555
10. Tsang TK, Wu P, Lin Y, Lau EHY, Leung GM, Cowling BJ. Effect of changing case definitions for COVID-19 on the epidemic curve and transmission parameters in mainland China: a modelling study. The Lancet Public Health. 2020;5(5). doi:10.1016/S2468-2667(20)30089-X
11. WHO Coronavirus Disease (COVID-19) Dashboard [database on the Internet]2020. Available from: https://covid19.who.int/.
12. Salje H, Kiem CT, Lefrancq N, et al. Estimating the burden of SARS-CoV-2 in France. Science. 2020;369(6500):208-11. doi:10.1126/SCIENCE.ABC3517
13. Fung IC-H, Hung YW, Ofori SK, Muniz-Rodriguez K, Lai P-Y, Chowell G. SARS-CoV-2 Transmission in Alberta, British Columbia, and Ontario, Canada, January 1-July 6, 2020. medRxiv. 2020:2020.07.18.20156992. doi:10.1101/2020.07.18.20156992
14. Cori A, Ferguson NM, Fraser C, Cauchemez S. A New Framework and Software to Estimate Time-Varying Reproduction Numbers During Epidemics. American Journal of Epidemiology. 2013;178(9):1505-12. doi:10.1093/AJE/KWT133
15. Muniz-Rodriguez K, Fung I, Ferdosi S, et al. Severe Acute Respiratory Syndrome Coronavirus 2 Transmission Potential, Iran, 2020. Emerging infectious diseases. 2020;26(8). doi:10.3201/eid2608.200536
